# Pathogenic polyglutamine expansion length correlates with polarity of the flanking sequences

**DOI:** 10.1186/1750-1326-9-45

**Published:** 2014-11-06

**Authors:** Meewhi Kim

**Affiliations:** Department of Physiology, UT Southwestern Medical Center, Dallas, TX 75390 USA; Laboratory of Molecular Neurodegeneration, St Petersburg State Polytechnical University, St Petersburg, 195251 Russian Federation

**Keywords:** Polyglutamine disorders, Primary sequence analysis, Proteasome, Huntingtin, Ataxin

## Abstract

**Background:**

Polyglutamine (polyQ) repeat expansion within coding sequence of a soluble protein is responsible for eight autosomal-dominant genetic neurodegenerative disorders. These disorders affect cerebellum, striatum, basal ganglia and other brain regions. The pathogenic polyQ-expansion threshold in these proteins varies from 32Q to 54Q. Understanding the reasons for variability in pathogenic polyQ threshold may provide insights into pathogenic mechanisms responsible for development of these disorders.

**Findings:**

Here we established a quantitative correlation between the polarity of the flanking sequences and pathogenic polyQ-expansion threshold in this protein family. We introduced an “edge polarity index” (^E^PI) to quantify polarity effects of the flanking regions and established a strong correlation between ^E^PI index and critical polyQ expansion length in this protein family. Based on this analysis we subdivided polyQ-expanded proteins into 2 groups – with strong and weak dependence of polyQ threshold on ^E^PI index. The main difference between members of the first and the second group is a polarity profile of these proteins outside of polyQ and flanking regions. PolyQ proteins are known substrates for proteasome and most likely mechanistic explanation for the observed correlation is that proteasome may have an impaired ability to process continuous non-polar regions of proteins.

**Conclusions:**

The proposed hypothesis provides a quantitative explanation for variability in pathogenic threshold among polyQ-expansion disorders, which we established to correlate with polarity of flanking regions. To explain these results we propose that proteasome is not efficient in processing continuous non-polar regions of proteins, resulting in release of undigested and partially digested fragments. If supported experimentally, our hypothesis may have wide implications for further understanding the pathogensis of polyglutamine expansion disorders.

## Findings

### Introduction

An expansion of CAG-repeat encoding continuous stretch of glutamines results in polyQ expansion in the context of several proteins. These polyQ expansions are responsible for nine genetic neurodegenerative disorders affecting cerebellum, striatum, basal ganglia and other brain regions [1, 2]. These disorders include Huntington’s disease (HD), Dentatorubral-pallidoluysian atrophy (DRPLA), Spinal Bulbar Muscular atrophy (SBMA), and spinocerebellar ataxias (SCAs) 1, 2, 3, 6, 7 and 17 [3–8] (Table 1). In the case of SCA6, the polyQ expansion occurs in the context of a membrane protein – a pore forming subunit of Ca_V_2.1 voltage-gated calcium channel. For other 8 disorders the polyQ expansion occurs in the context of soluble proteins localized to the cytosol or the nucleus of neuronal cells. Each of these disorders has a characteristic pathogenic threshold for minimal polyQ expansion that causes disease [3–8]. Once this pathogenic threshold is exceeded, the age of disease onset is inversely proportional to polyQ expansion length [1]. With exception of the polyQ-expanded region, these proteins do not share other common sequence motifs or similar biological functions. All of these disorders are transmitted in an autosomal dominant fashion and the most likely reason for pathology is accumulation of polyQ-expanded proteins or protein fragments in cells. PolyQ-expanded proteins are able to interfere with multiple neuronal signaling pathways, such as protein aggregation, apoptotic cascade, calcium signaling, proteasomal dysfunction, gene transcription and many others [9–16]. The interference with one or several of these signaling cascades eventually results in neuronal dysfunction and death, causing the disease. Pathogenic polyQ-expansion threshold (Q_th_) of soluble proteins in this gene family varies from 32Q (for SCA2) to 54Q (for SCA3) (Table 1). The reason for variability of the pathogenic threshold among these proteins is not understood. To explain this phenomenon, we performed comparative sequence analysis of the eight soluble polyQ-expanded proteins. Based on results of this quantitative analysis we established a correlation between the polarity of the flanking sequences and pathogenic polyQ-expansion threshold. To explain these results we propose that proteasome is not efficient in processing continuous non-polar regions of proteins, resulting in release of undigested and partially digested polyQ fragments.

**Table 1:** **PolyQ-expansion diseases** [1]**,** [2]**,** [17]

Disease	Protein	Localization	Q _th_	^N^PI	^C^PI	^E^PI	Neuropathology
**HD**	Htt	Cytoplasm	35	7.925	-2.047	5.878	Striatum, Cortex
**SBMA**	AR	Nucleus, Cytoplasm	38	-3.658	19.475	15.817	Spinal cord, Brain stem
**DRPLA**	ATN1	Nucleus, Cytoplasm	49	53.530	36.706	90.236	Cerebellum, Basal ganglia
**SCA1**	Atxn1	Nucleus	39	35.109	25.323	60.432	Cerebellum, Brain stem
**SCA2**	Atxn2	Cytoplasm	32	13.614	-2.222	11.392	Cerebellum, Brain stem
**SCA3**	Atxn3	Nucleus, Cytoplasm	54	20.552	12.979	33.531	Ventral pons, Substantia nigra
**SCA6**	CACN1A	Plasma Membrane	20	-	-	-	Cerebellum
**SCA7**	Atxn7	Nucleus, Cytoplasm	37	19.309	-2.047	17.262	Cerebellum, Brain stem, Spinal cord
**SCA17**	TBP	Nucleus	47	26.816	-3.758	23.058	Cerebellum, Striatum

*Footnote:* Q_th_ - pathogenic polyQ-expansion threshold; ^N^PI - amino terminal polarity index; ^C^PI - carboxy-terminal polarity index; ^E^PI - total edge polarity index.

### Methods

#### Calculation of protein polarity profiles and polarity edge indexes

The Zimmerman Polarity index [18] for each protein was calculated by using ProtScale software package [19] with the following options: window size: 9, Relative weight of the window edges compared to the window center: 100%, Weight variation model: linear. The polarity edge index was derived by calculating change in polarity on the edges of polyQ sequence induced by the flanking regions. The amino terminal polarity index (^**N**^PI) and carboxy-terminal polarity index (^**C**^PI) are shown in Table 1. The total edge polarity index (^**E**^PI) was calculated as a sum of ^**N**^PI and ^**C**^PI indexes.

### Results

#### Primary protein-sequence comparison of polyQ-expanded protein family

**Figure 1:**
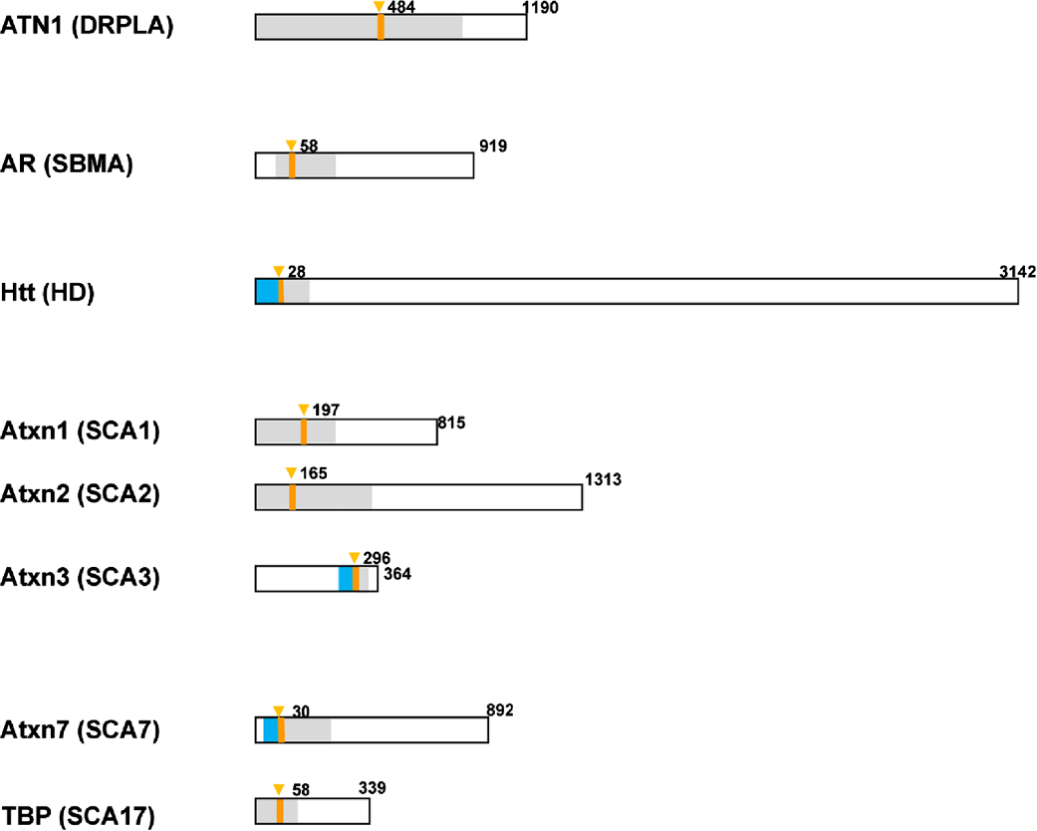
**The family of soluble polyQ-expanded proteins.** Schematic diagram of 8 soluble polyQ-expanded proteins. The last amino acid in each protein is numbered. The polyQ region is shown by an orange bar. The first residue of polyQ stretch is indicated by down triangle and numbered. The areas shaded by grey correspond to the loop regions based on secondary structure predictions. Predicted alpha-helical regions are shown by blue color.

To determine potential sources of variability of pathogenic polyQ expansion threshold, we compared protein sequences of 8 soluble proteins in polyQ-expanded gene family (Figure 1). The CACNA1 protein responsible for SCA6 was excluded from our analysis because CACNA1 is the only membrane protein in the family and because the polyQ-expansion threshold for SCA6 (Q_th_ = 20) is much lower than those for all other polyQ-expansion disorders (Q_th_ > 35). Most likely the pathogenic mechanism of SCA6 is distinct from all other disorders and directly related to changes in function or expression of Ca_V_2.1 voltage-gated Ca^2+^ channels [6]. The soluble members of polyQ-expanded protein family differ widely in size – from TBP (339 aa long) to Htt (3142 aa long) (Figure 1). The polyQ region (shown by orange bar) is located in the amino terminal portion of a protein (AR, Htt, Atxn1, Atxn2, Atxn7, TBP), in the central region of a protein (ATN1) or in the carboxy-terminal portion of a protein (Atxn3). Secondary structure predictions suggest that polyQ stretches in these proteins are flanked by a loop (grey) or α-helical (blue) secondary structure. The presence of α-helical flanking amino-terminal regions in Htt and Atxn3 proteins have been confirmed by crystallographic analysis ([20, 21] and Meewhi Kim, unpublished observations). Crystallographic information for other polyQ-expanded proteins is not presently available. This initial analysis has not revealed any specific protein sequence pattern that could explain the variation in pathogenic polyQ-expansion threshold among the protein family (Table 1).

#### Sequence polarity and proteasomal processing of protein

**Figure 2:**
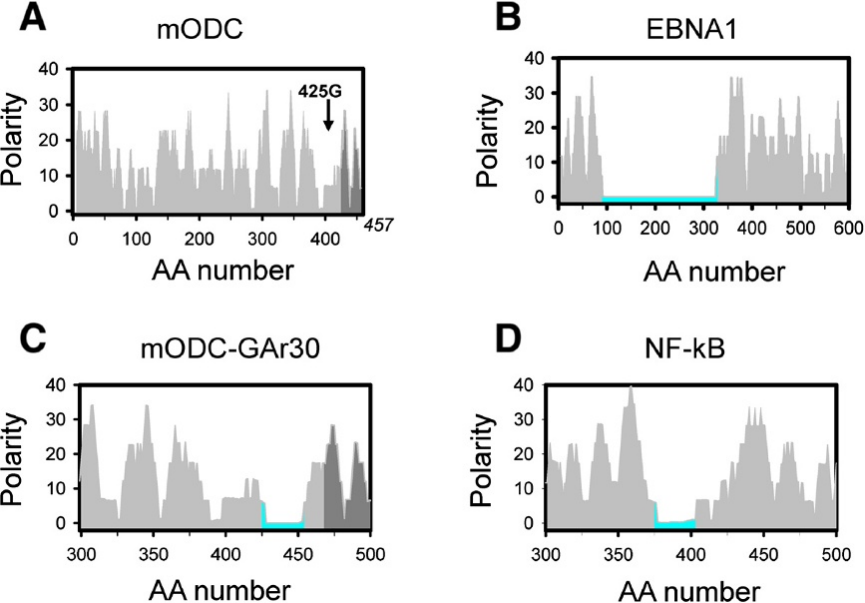
**Polarity profiles of model proteasomal substrates.** Calculated polarity profiles are shown for mODC **(A)**; EBNA1 **(B)**; ODC-GAr30 **(C)**; NF-kB **(D)**. Extended low polarity regions are colored with blue on panels **B**, **C**, **D**. In ODC-GAr30 construct the GAr30 repeat sequence was inserted in position 425G of mODC protein (shown on panel **A**).

In cells, fragments of polyQ-expanded proteins are often found within ubiquitin-positive inclusions. These findings suggest that polyQ expansion may influence degradation of these proteins by proteasome [22–25]. Thus, we reasoned that the variability of pathogenic polyQ threshold may be due to variable effects of polyQ-flanking regions on proteasomal processing of these proteins. To better understand potential effects of protein sequence on proteasomal processing, we analyzed primary sequences of several model proteins which have been used previously as substrates for studies of proteasomal function. These proteins include mouse ornithine decarboxylase (mODC), which is degraded rapidly by the proteasome [26]. These proteins also included Epstein-Barr virus nuclear antigen1 (ENBA1) and Nuclear Factor kappa-light-chain-enhancer of activated B cells (NF-kB), both of which are extremely poor substrates for proteasomal degradation [27, 28]. Proteasomal processing of these proteins is determined by their amino acid sequences. It has been previously suggested that the resistance to proteasomal processing is related to low sequence complexity of ENBA1 and NF-kB proteins [27, 28]. We reasoned that sequence polarity may also affect proteasomal processing of these proteins. To evaluate this idea, we performed analysis of polarity profiles of these model proteasomal substrates. For mODC protein the polarity profile oscillates between 0 and 40 with periodicity between 5 to 20 amino acid residues through entire protein (Figure 2A). Such polarity profile appears to be optimal for proteasomal processing. In contrast, ENBA1 protein contains 250 amino acid long Glycine-Alanine repeats (GAr) with polarity close to 0 (Figure 2B). Similarly, NF-kB protein contains 30 amino acid long Glycine rich repeat region (GRR) of low polarity (Figure 2D). This analysis suggests that the presence of extended low polarity region is incompatible with proteasomal processing of a substrate. Consistent with this interpretation, the insertion of a 30 amino acid long GAr repeat within mODC protein (ODC-GAr30) (Figure 2C) halted the proteasomal processing of the fusion protein, resulting in accumulation of undigested fragments of mODC-GAr30 in cells [29]. Based on this analysis we proposed that the presence of intermittent high/low polarity regions facilitates proteasomal degradation of proteins and the presence of extended low polarity regions impairs it due to premature release of incompletely digested substrate. This hypothesis is consistent with studies of dihydrofolate reductase (DHFR) degradation by proteasome [30]. Consistent with our hypothesis, insertion of low polarity stretches of 37S, 37N, or 37G aminoacids impaired ability of proteasome to process DHFR substrate in these experiments and lead to accumulation of partially digested products [30].

#### Polarity of polyQ-flanking regions and pathogenic polyQ threshold

The expanded polyQ stretch corresponds to an extended low polarity sequence. As discussed above, we propose that such a sequence is a poor substrate for proteasomal processing. This argument may explain the accumulation of undigested ubiquitinated fragments of polyQ-expanded proteins in cells [23, 24, 31–36]. We further reasoned that the pathogenic threshold of polyQ expansion may be related to potential influence of flanking regions on proteasomal degradation of naked polyQ sequence. Specifically, if polyQ sequence is surrounded by highly polar flanking regions, then these regions can increase the effective polarity on the edges of polyQ stretch, promoting proteasomal processing. On the other hand, if polyQ region is flanked by low polarity regions, then the polarity of polyQ sequence edges remains low, impairing proteasomal processing. This hypothesis predicts that a polyQ sequence embedded within polar flanking sequences must have longer expansion to reach pathogenic threshold than the polyQ sequence embedded within low polarity flanking sequences.

**Figure 3:**
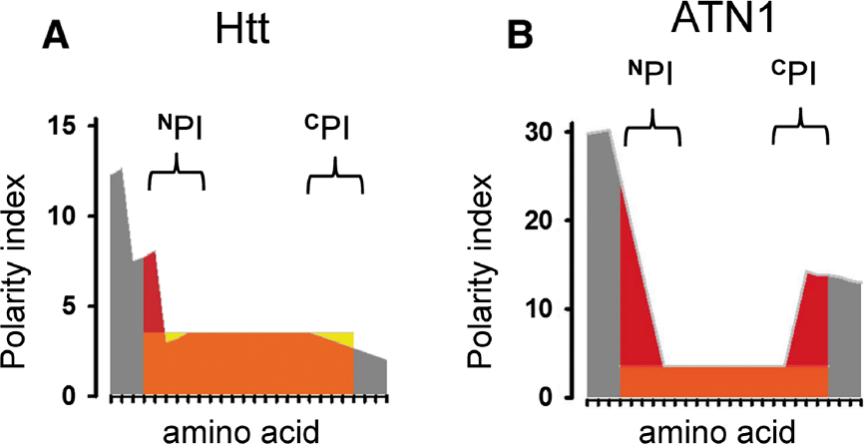
**Calculation of Edge Polarity Index.** The polarity profile is shown for the polyQ stretch and immediate flanking regions for Htt **(A)** and ATN1 **(B)**. The polarity index of naked polyQ stretch (3.5) is shown by orange. The polarity index of flanking regions are shown by grey. Effective increase of polarity index on the edges of polyQ sequence is shown by red. Effective decrease of polarity index on the edges of polyQ sequence is shown by yellow. ^N^PI and ^C^PI indexes were calculated by integrating edge polarity effects at amino-terminal and carboxy-terminal ends of polyQ sequence.

To quantitatively test this hypothesis, we performed calculations of Edge Polarity Index (^**E**^PI) for each of the soluble polyQ-expanded proteins (Figure 1). The procedure used for these calculations is illustrated for Htt and ATN1 proteins on Figure 3. The polarity index of polyQ sequence (orange) is 3.5. Such value is indeed achieved in the middle of the polyQ sequence, away from the edges (Figures 3A,B). However, at the edges of polyQ sequence the polarity index deviates from the value of 3.5 due to the presence of the flanking regions (grey). If polarity of amino acids in the flanking regions exceeds 3.5, then the effective polarity on the edges of polyQ sequence is increased (shown by red color). However, if polarity of amino acids in the flanking regions is lower than 3.5, then effective polarity on the edges of polyQ sequence is reduced (shown by yellow color). To quantify these results, we calculated an integral change in polarity on the amino-terminal end (^**N**^PI) and carboxy-terminal end (^**C**^PI) of the polyQ sequence. In these calculations a value of 3.5 was subtracted from the generated polarity index for each glutamine residue. The edge polarity index (^**E**^PI) was calculated as a sum of ^**N**^PI and ^**C**^PI values. Calculated ^**N**^PI, ^**C**^PI and ^**E**^PI values are presented in Table 1 for each of the soluble polyQ-expanded proteins. The ^**E**^PI index for Htt is 5.878, one of the lowest in this protein family (Table 1). This is due to the presence of low polarity flanking sequence at the amino-termini of polyQ region and the presence of non-polar proline stretch at the carboxy-termini of the polyQ region (Figure 3A). In contrast, ATN1 has ^**E**^PI index of 90.236, the highest in this protein family (Table 1). This is due to the presence of highly polar flanking regions on both amino-terminal and carboxy-terminal ends of polyQ sequence (Figure 3B).

**Figure 4:**
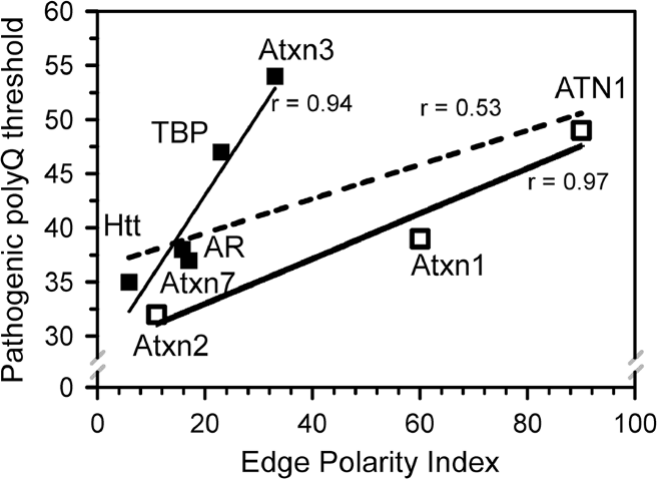
**Correlation between pathogenic polyQ expansion threshold and Edge Polarity Index.** Pathogenic polyQ-expansion threshold is plotted versus ^E^PI index for each of the 8 soluble polyQ-expanded proteins. Linear fit for all 8 data points is shown by a dashed line (r = 0.53). The liner fit for group 1 (filled squares, r = 0.94) and group 2 (open squares, r = 0.97) members is shown by solid lines.

In order to test our hypothesis we aimed to establish the relationship between minimal pathogenic polyQ threshold and the polarity effects of the flanking sequences. When the length of polyQ pathogenic threshold was plotted versus ^**E**^PI for each polyQ-expanded protein, we observed a significant correlation (Figure 4, dashed line, r = 0.53). The quality of the fit was improved significantly after separation of the eight soluble polyQ-expanded proteins into 2 groups. The first group includes Htt, Atxn7, AR, TBP and Atxn3 (Figure 4, filled squares, r = 0.94). For this group polyQ pathogenic threshold increases as a strong function of the ^**E**^PI index (Figure 4). The second group includes Atxn2, Atxn1, and ATN1 (Figure 4, open squares, r = 0.97). For these proteins polyQ pathogenic threshold increases as a weak function of the ^**E**^PI index (Figure 4).

#### Pathogenic polyQ threshold and influence of protein context

**Figure 5:**
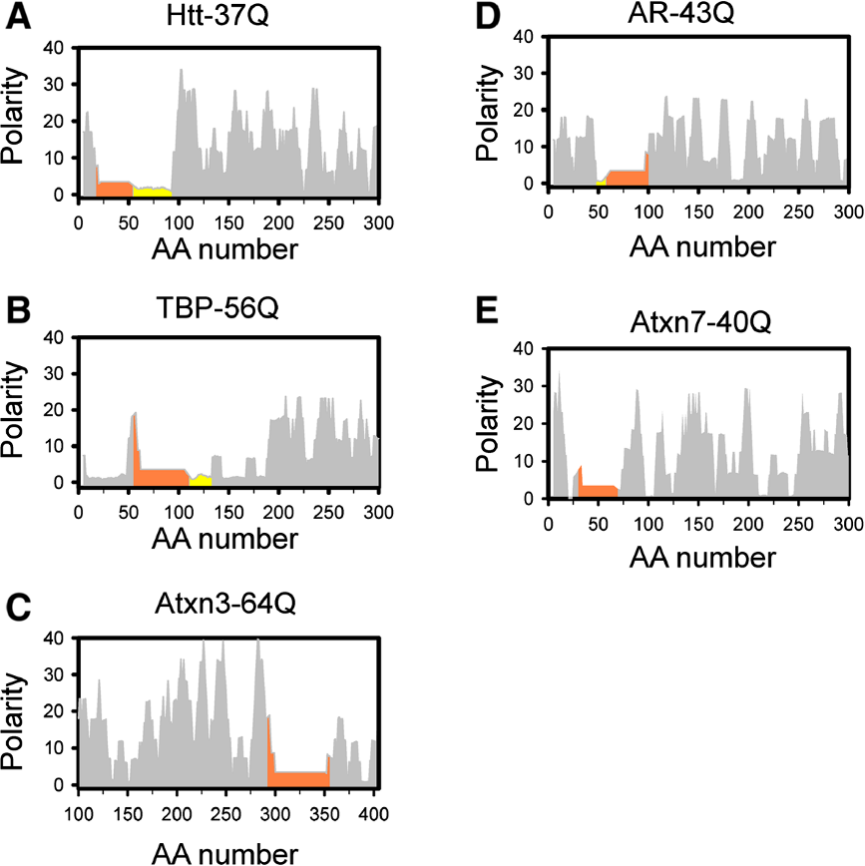
**Polarity profiles of Group 1 polyQ-expanded proteins.** Polarity index profiles are shown for Htt-37Q **(A)**; TBP-56Q **(B)**; Atxn3-64Q **(C)**; AR-43Q **(D)**; Atxn7-40Q **(E)**. PolyQ region in each protein is shown by orange. The proline-rich regions are shown by yellow. Full-length protein profile is shown for TBP protein. The profiles for 300 amino-acid long protein fragments are shown for other proteins.

**Figure 6:**
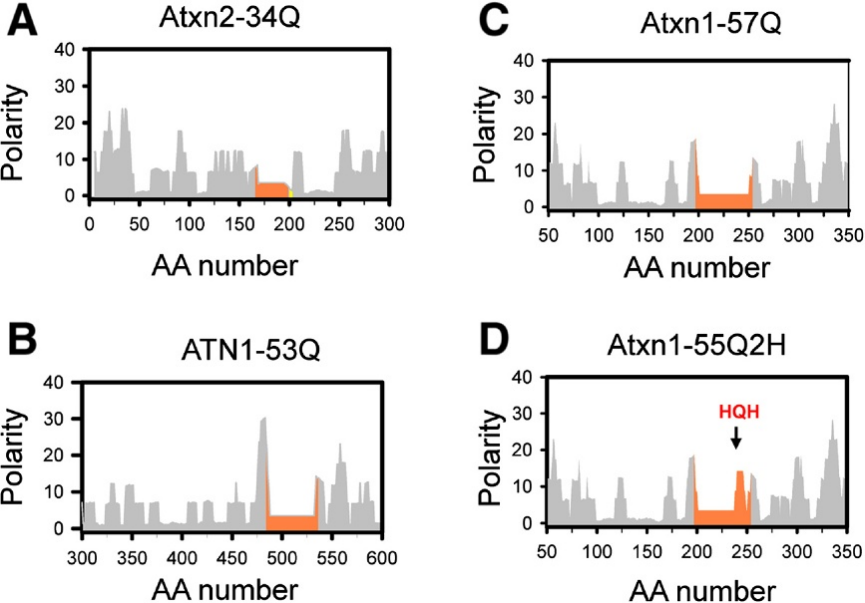
**Polarity profiles of Group 2 polyQ-expanded proteins.** Polarity index profiles are shown for Atxn2-34Q **(A)**; ATN1-53Q **(B)**; Atxn1-57Q **(C)** and Atxn1-55Q2H **(D)**. PolyQ region in each protein is shown by orange. The profiles for 300 amino-acid long protein fragments are shown for each protein. The site of HQH insertion is shown by arrow on panel D.

To understand the difference between the proteins in the first and second groups we analyzed the polarity profiles for each of these proteins in more detail. Whole protein sequence was analyzed for TBP protein, the shortest member in this family. For the remaining 7 proteins we focused on the 300 amino-acid region that included polyQ sequence. The main reason to focus on just 300 amino-acid region is that release of undigested polyQ-containing fragment has most important implications for toxicity of these proteins in cells when compared to remaining portions of these proteins. Calculated polarity profiles for 5 members of the first group are shown on Figure 5. Htt protein contains an extended low-polarity proline-rich flanking region on carboxy-terminal side of the polyQ stretch (Figure 5A, shown in yellow). Due to the presence of this low polarity region pathogenic polyQ threshold for HD (Q_th_ = 35) is one of the shortest in this family of proteins (Table 1). With the exception of polyQ and flanking regions the rest of the Htt N-terminal region is composed of regular high-low polarity residues. The polyQ-flanking domains in TBP are a polar peak at N-terminal and a short non-polar proline-rich domain at C-terminal (Figure 5B). Atxn3 contains regular polar flanking regions at both sides (Figure 5C). Sequences of AR and Atxn7 are composed of residues with regular polar regions (Figures 5D,E). Therefore, with exception of the polyQ stretches and flanking regions, all members of this group are composed of regular low-high polarity sequences (Figures 5A-E). We suggest that such sequences are good substrates for proteasomal degradation. The polarity plots for 3 members of the second group (Atxn2, Atxn1, ATN1) are shown in Figure 6. These 3 proteins are composed primarily of low polarity regions (Figures 6A-C). We suggest that such sequences are poor substrates for proteasomal degradation. Based on this analysis we propose that the difference between the members of the first and the second groups come from the overall polarity profiles of these proteins beyond polyQ stretch and immediate flanking sequences.

Interestingly, each member of the first group has been reported to be associated with proteasome in biochemical experiments. In some cases association with proteasome occurred via ubiquitinated form of the protein - such as AR [37, 38], Atxn3 [39, 40], or Htt [41, 42]. In some cases association with proteasome did not require ubiquitination, such as for Atxn7 [43, 44] and for TBP [45–47]. In contrast, members of the second group have not been reported to associate with proteasome in biochemical experiments. The only known interaction is proteasomal association of Atxn1 that is mediated by HSP/CHIP [48, 49] and requires partial unfolding of Atxn1 to be initiated. Although correlative, this argument further suggests that the members of the first group are better substrates for proteasomal degradation than the members of the second group.

#### Effects of Histidine insertion

A unique clinical case provides an indirect support to our hypothesis. A Japanese SCA1 patient was discovered to have an insertion of 2 His residues within polyQ stretch, resulting in sequence Q_45_HQHQ_10_ [50]. An expected age of disease onset for a typical SCA1-58Q patient is 22 years of age. In contrast, the SCA1-Q_45_HQHQ_10_ patient displayed first symptoms of disease at the age of 50 [50]. In addition, the brain stem atrophy of this patient was much milder than expected for a typical SCA1 patient with similar repeat length [50]. What is an explanation for dramatic protective effects of His insertion? Biophysical studies [51–53] and our own crystallographic experiments [21] suggested that insertion of His has minimal effect on secondary structure of polyQ region. Thus, it is not likely that insertion of 2 His residues disrupted the “toxic conformation” of the 58Q stretch. However, insertion of 2 His residues is expected to introduce a polarity peak within polyQ sequence. Indeed, the polarity profile of Atxn1-55Q2Н (Q_45_HQHQ_10_) contains a significant polar peak (Figure 6D). We propose that such polar insertion enhances proteasomal processing of His-containing protein. As a result, “effective” low polarity polyQ region is shortened to approximately 36Q (Figure 6D), which is consistent with the very mild clinical phenotype of this particular patient [50].

### Conclusion

In this paper we established a quantitative correlation between the polarity of the flanking regions and the pathogenic polyQ expansion threshold for the soluble polyQ-containing proteins. The quantitative analysis enabled us to divide soluble polyQ-expanded proteins into 2 groups – with strong and weak dependence of polyQ threshold on the polarity of the flanking regions. The main difference between members of the first group (Htt, Atxn7, AR, TBP, Atxn3) and the second group (Atxn2, Atxn1, ATN1) is related to polarity profile of remainder of these proteins. All members of the first group composed of regular low-high polarity sequences, whereas members of the second group are composed primarily of low polarity sequence regions. PolyQ proteins are known substrates for proteasomal degradation. We analyzed experiments performed with the model proteasomal substrates and concluded that proteasome has impaired ability to process continuous non-polar regions of proteins. We propose that polarity of flanking regions may have an important modulatory effects on ability of proteasome to process continuous polyQ stretches, resulting in accumulation of polyQ-expanded proteins and partially digested protein fragments in cells. Such proteins can then exert “toxic gain of function” effects by interfering with essential neuronal signaling pathways. These ideas need to be tested experimentally. However, indirect support to our hypothesis is provided by partial protective effects of His insertion within polyQ stretch of SCA1 patient, which has a significant effect on polarity profile of polyQ stretch. We propose that such polar insertion can facilitate proteasomal degradation of polyQ-expanded Ataxin 1, which may explain less severe pathology in these patients.
